# SEVA 2.0: an update of the Standard European Vector Architecture for de-/re-construction of bacterial functionalities

**DOI:** 10.1093/nar/gku1114

**Published:** 2014-11-11

**Authors:** Esteban Martínez-García, Tomás Aparicio, Angel Goñi-Moreno, Sofía Fraile, Víctor de Lorenzo

**Affiliations:** Systems Biology Program, Centro Nacional de Biotecnología (CNB-CSIC), 28049 Cantoblanco-Madrid, Spain

## Abstract

The *Standard European Vector Architecture* 2.0 database (SEVA-DB 2.0, http://seva.cnb.csic.es) is an improved and expanded version of the platform released in 2013 (doi: 10.1093/nar/gks1119) aimed at assisting the choice of optimal genetic tools for de-constructing and re-constructing complex prokaryotic phenotypes. By adopting simple compositional rules, the SEVA standard facilitates combinations of functional DNA segments that ease both the analysis and the engineering of diverse Gram-negative bacteria for fundamental or biotechnological purposes. The large number of users of the SEVA-DB during its first two years of existence has resulted in a valuable feedback that we have exploited for fixing DNA sequence errors, improving the nomenclature of the SEVA plasmids, expanding the vector collection, adding new features to the web interface and encouraging contributions of materials from the community of users. The SEVA platform is also adopting the Synthetic Biology Open Language (SBOL) for electronic-like description of the constructs available in the collection and their interfacing with genetic devices developed by other Synthetic Biology communities. We advocate the SEVA format as one interim asset for the ongoing transition of genetic design of microorganisms from being a trial-and-error endeavor to become an authentic engineering discipline.

## INTRODUCTION

Since its release in 2013 (1) the Standard European Vector Architecture Database (SEVA-DB) has provided the Molecular Microbiology and Microbial Biotechnology communities with a curated catalog of molecular tools for advanced genetic engineering of Gram-negative bacteria. One unique feature of such an inventory is that its components are subject to a concise, minimalist and standardized format and nomenclature that is motivated by the tenets of Synthetic Biology (2,3). A second distinctive quality of the SEVA collection is that the database is linked to a material repository of vectors and constructs (1,4) which can be requested by academic users-to-be under an Open Access policy (some restrictions may apply to industrial users, see below). Finally, every material of the SEVA list has been thoroughly checked for functionality, and potential users are encouraged to immediately report any problem or divergence from what is expected. This makes the database and its cognate vector repository to reach a professional standard that is not found in other much larger, but not curated collections of biological parts, e.g. http://parts.igem.org (see reference (5) for a critical view).

During the short time (<2 years) that the database (http://seva.cnb.csic.es) has been in operation, it has received >470 vector requests from 25 countries and the primary publication describing the platform (1) has had >40 citations thus far in the academic literature. Also, more plasmids have been generated not only in our own Laboratory but also in others’, which follow the SEVA standard to different degrees and which have been kindly handed over to the DB curators for maintenance and possible distribution. In the meantime, new standards and principles stemming from Systems and Synthetic Biology (6–8) have emerged which intersect with the original agenda of the SEVA initiative. All this has resulted in a favorable opportunity to revisit the organization and contents of the first database for both updating the record of available materials and expanding the utilities in new directions. As before, SEVA-DB 2.0 is grounded on a basic collection of synthetic, interchangeable and reusable functional modules: origins of replication (including narrow and broad-host-range), antibiotic markers and cargoes (mostly expression systems and reporter genes) that are punctuated by terminators and non-used insertion sites. These have been enriched with new functional segments and rigorous descriptions and representations of the constructs (i.e. by incorporating the SBOL standard) that expand their connectivity to other SynBio platforms. As shown below, this has resulted in a considerably improved DB that will hopefully keep on easing biosystems engineering beyond laboratory applications.

## DATABASE DESCRIPTION

### Database organization

As was the case with its former version, the updated SEVA database (SEVA-DB 2.0, http://seva.cnb.csic.es) serves primarily as an annotated and information-rich index of functional DNA sequences and constructs that are available to the community. The platform maintains the simple architecture of its predecessor, which consists of a relational database as the data storage layer, a series of modules that are hosted by an application server and a web-based presentation layer with an explicit set of standards that apply to all constructs and can in most cases send the vector sequences to a remote analysis server. To this end, the corresponding DNA sequences of each plasmid are accessible either through a link to permanent GenBank numbers or to an interim .gbk file. A subset of DNA sequences of the vector collection can be further exported through a direct link to the plasmid map viewer of LabGenius (www.labgeni.us), a platform that allows visualization of all functional parts of the vector along with a large and user-friendly collection of utilities, e.g. virtual restriction digests and indications for sequencing primers.

**Figure 1.**
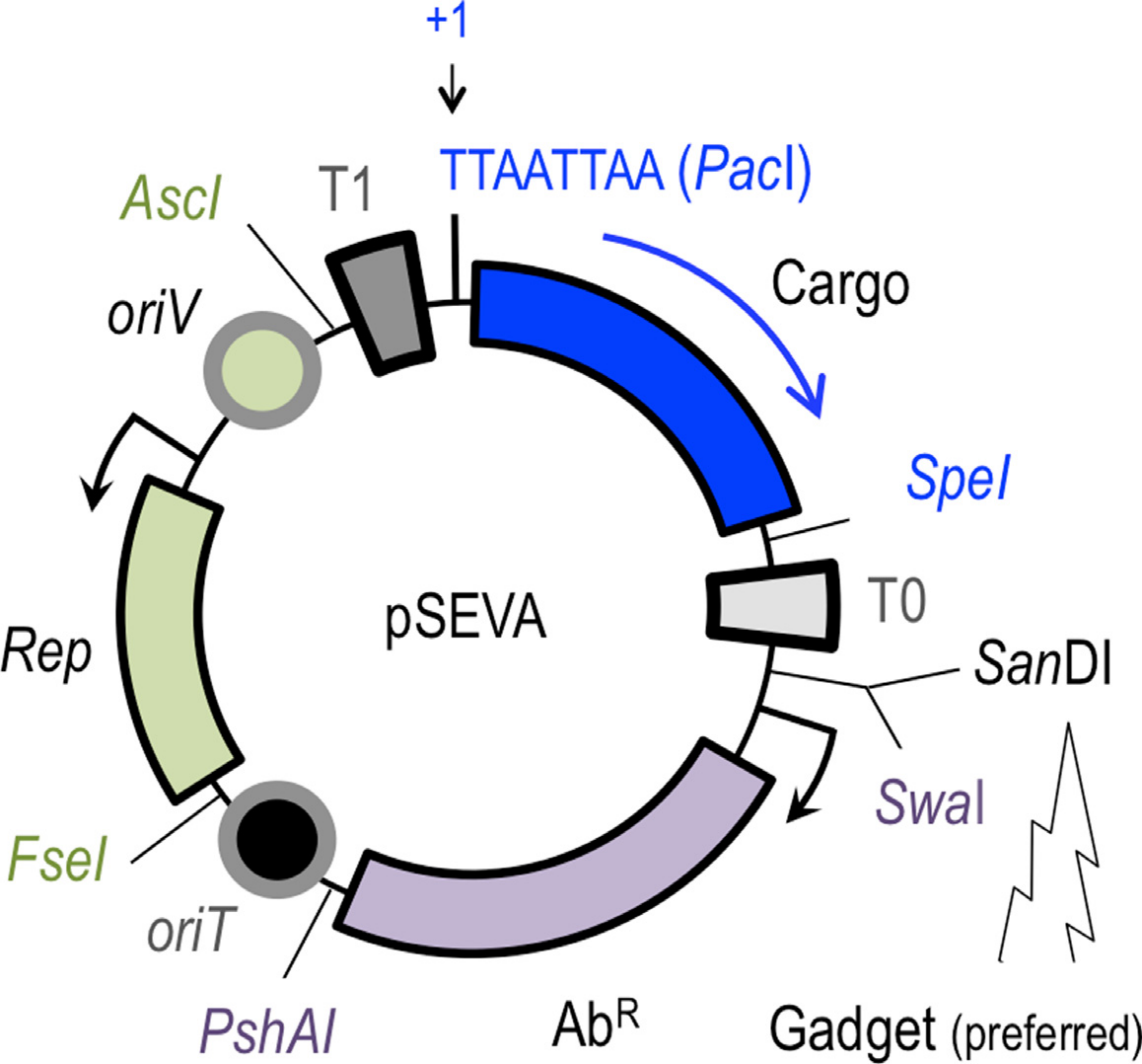
The formatted structure of SEVA plasmids. SEVA vectors are shaped by three basic modules: a cargo (blue), a plasmid replication origin (green) and an antibiotic marker (magenta). Restriction sites that punctuate the boundaries between modules in all constructs are indicated. Note the numbering position +1 of the DNA sequence is the first T of the unique *Pac*I site. The directionality of the stronger transcription flow in the genetic device engineered in the cargo and the preferred site for inserting functional gadgets are indicated. See http://seva.cnb.csic.es for details.

Once faced with the general structure of the SEVA vectors (Figure 1), users can easily decide what is the best configuration of replication origin, antibiotic resistance and business segment (cargo) that serves better their specific purposes. The visual web interface has been reshaped to make it more intuitive and user-friendly, and it also contains more entries/tabs, the contents of which are explained below. The most salient changes or improvements of the SEVA-DB 2.0 in respect to its earlier form are examined in the following paragraphs. Any other features of the platform not explicitly indicated as a change can be safely considered to be identical to the first complete description of the system as published in (1). The web curators regularly update new acquisitions and information and, to the best of their abilities, aim at maintaining an interactive and dynamic platform in real time. Most clones are readily constructed and ready for release upon request.

### The layout of SEVA plasmids

The shared architecture of all SEVA vectors is indicated in Figure 1. Each of them is composed of six functional modules, three of which are variable (origin of replication, selection marker and cargo) while the other three are fixed connectors that provide the reference for the whole vector layout. The boundaries between each of the modules are set by unusual restriction sites. Two of the connector sequences span strong transcriptional terminators, while the third spans an origin of conjugative transfer (*oriT*) for mobilization of pSEVA plasmids among bacterial species. Note that the first T nucleotide of the *Pac*I restriction site that lies to the left of the cargo module as shown in Figure 1 is arbitrarily designated as position +1 of the clockwise DNA sequence coordinates of all plasmids of the collection. Also, notice that the major course of action of any device engineered in the cargo segment follows, by default, the direction *Pac*I → *Spe*I. In typical expression cargoes that include the gene for a signal-responsive transcriptional regulator (expressed through its own promoter) adjacent to its cognate target DNA the relative orientation of the two (or more) transcription flows should be such that the stronger promoter follows an orientation *Pac*I → *Spe*I. Finally, since the updated SEVA collection contains for the first time a functional insertion in one of the sites that punctuate the boundaries between modular segments we need to clarify what we formerly designated on passing as plasmid *gadgets* (1). These are dispensable DNA sequences that endow, however, a new utility or property to the basic frame of the vectors. The SEVA standard allows addition of up to six new gadgets of the kind, as this is the number of unusual (and unique) restriction sites that by default punctuate the intervening regions between the three primary modules (Figure 1). All of such sites are in principle permissive for insertions of functional extra sequences. However, the site *Swa*I (which is adjacent to another rare, restriction site for enzyme *San*DI; Figure 1) is preferred over the others, as it allows directional cloning. For example, the plasmid-stabilization *hok/sok* gadget (9; see below) was inserted at that site of the low-copy number plasmids pSEVA221 and pSEVA251 as a *San*DI-*Swa*I fragment (see plasmid list). Insertion of a gadget should not make the cognate site unusable: instead, it should recreate the same unique restriction sequences at the sides of the insert.

### Re-coding the catalog of pSEVA vectors

One of the original tenets of the SEVA collection was the designation of each of the vectors with a non-ambiguous numerical code, so that each construct is named as pSEVA followed by a multi-digit cipher. Each position of the code was expected to mean the type of antibiotic resistance marker, the class of origin of replication and the specific cargo module. For example, pSEVA111 means a plasmid that has an Ap^R^ marker gene, the narrow-host-range origin of replication R6K and the default SEVA polylinker. However, as the collection has grown well beyond the upper limit of the nine variants allowed for each functional module, we have created an extended code for the vector catalog that keeps the designation of existing plasmids, incorporates constructs generated in recent times and covers possible future necessities.

**Figure 2.**
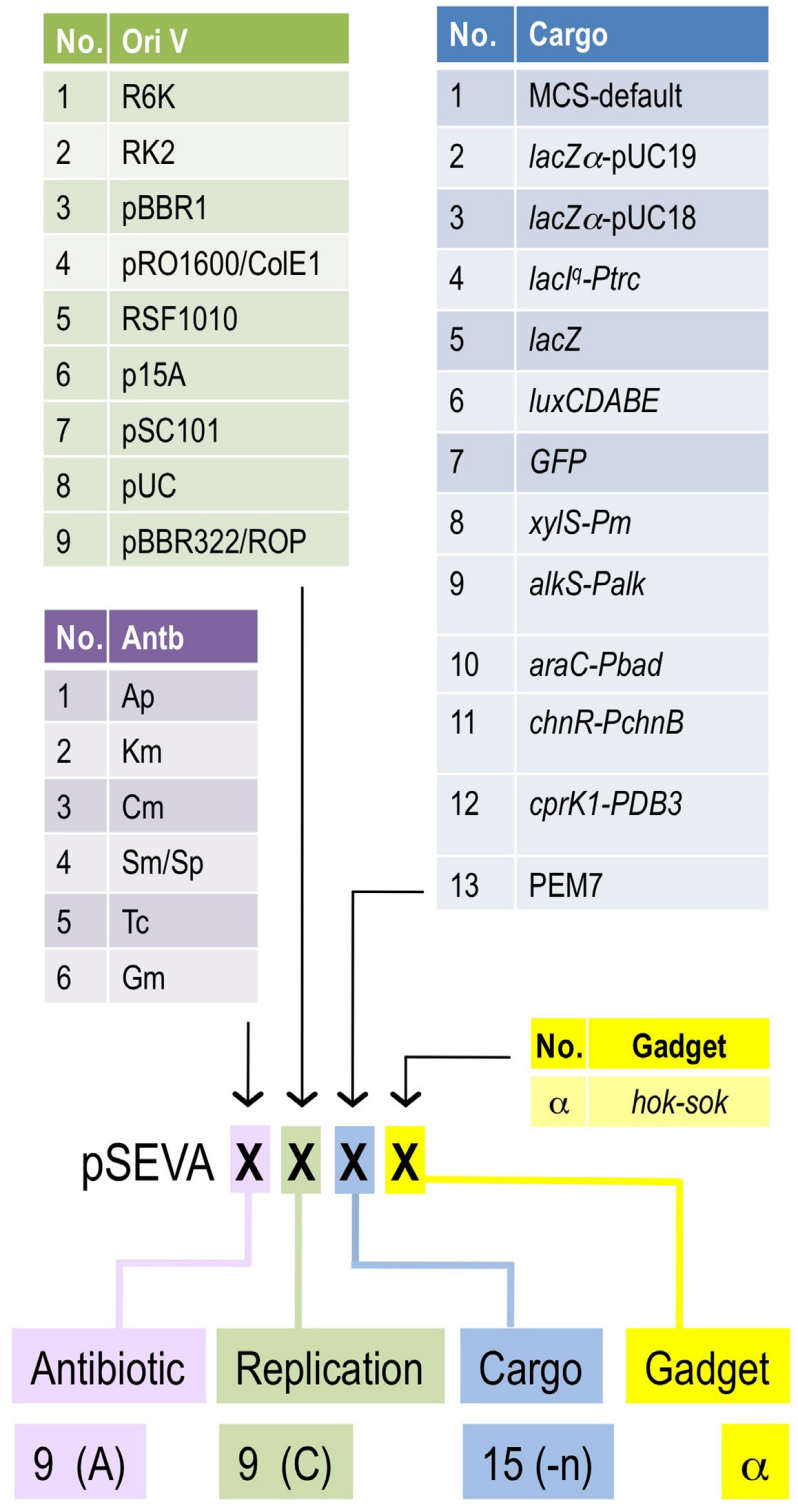
Expanded SEVA nomenclature. Vectors include four modules (antibiotic resistance marker, replication origin, cargo and gadget), which are represented in a code with four unequivocal positions. The *first* position is for antibiotic resistance markers, numbered 1 to 9 for the first nine and then 9B to 9Z for the next ones. The *second* position (the plasmid origin of replication) also receive a 1 to 9 code for the first variants and 9B to 9Z for those that follow. The cargo (*third* position), each cargo is assigned a sole number 1 to *n*, but the figure can be added with a capital letter in cases of variants of the same module. The *fourth* position is kept for the gadgets, which receive Greek letters (α to ω). See text for rationale and detailed explanation.

As shown in Figure 2, we consider each plasmid to be composed of four functional modules (antibiotic resistance marker, replication origin, cargo and gadget), which correspond to a code of four unequivocal positions in a cipher. Each position can have one or more numeric symbols as follows. The *first* position is for the antibiotic resistance marker, the first series of which received a sole numeric code (1 to 9). Beyond that number, a capital letter will be added to ‘9’, starting with an A (9A) and following with 9B, 9C etc. We avoid increasing the numeral beyond 9, as this may cause confusion with the rest of the code. It is unlikely that the new antibiotic resistances will exceed the capacity of such a numbering. Similarly, for the *second* position, the first nine origins of replication receive a sole numeric code 1 to 9. As before, beyond that figure, a capital letter will be added to ‘9’, starting with an A (9A) and following with 9B, 9C etc. The number of novel origins of replication is implausible to surpass that account. In the *third* position, each cargo is assigned a sole ordinal numeric code 1 to *n* (*n* being unlimited). This is because the number of cargoes will surely grow much faster than the other functional modules and thus this position of the code is not to be restrained. In the cases where there is a variant of the same cargo type (e.g. a new version of a promoterless fluorescent protein gene) we then add a capital letter to the numeral. For example, 7R is a variant of cargo #7 (GFP) that encodes the gene of the mCherry protein. Finally, the *fourth* position is for the gadgets, which are designated by lower case Greek letters (α to ω). This gives 24 possibilities that in case of completion can similarly be extended with capital Greek letters. For example: a pSEVA code 9C-9E-13-α (abridged: 9C9E13α) means a vector with a 9C antibiotic resistance marker, a 9E origin of replication, bearing cargo #13 and endowed with α gadget. To facilitate the coding and de-coding of the different plasmid ciphers, the SEVA web page includes a tab for generating vector codes and, if such a code is available, to disclose the structure and components of the cognate plasmid to potential users. Since the release of the 1.0 version of the database, the collection has increased the number of origins of replication, cargoes and gadgets (see plasmid list), while the antibiotic resistance markers have remained at the figure of 6.

### De-bugging and enlarging the canonical SEVA-DB 2.0 collection

One of the most significant—if inconspicuous change of the plasmid compendium 2.0 in respect to the initial vector list has been the fixing of an early assembly mistake that was propagated through all pSEVA plasmids that had an R6K origin of replication (i.e. all those with a ‘1’ digit in the second position of the code). Short after the release of the DB, one user alerted us that the GenBank sequences of the plasmids with such an *oriV* R6K had two *Asc*I sites flanking this functional segment. Once this observation was verified, we deleted 8 bp (5'GGCGCGCC3') of the sequence adjacent to the unique *Fse*I site (Figure 1; coordinates 1583–1590 of the unamended pSEVA111) in each of the 17 plasmids that were affected by the problem. The amended plasmids of the 2.0 DB (which maintain their former and now correct name) follow rigorously the SEVA standard. In addition to these, the collection has at this point 32 new standardized vectors that either resulted from new combinations of existing modules or constitute fresh contributions to the list. These last include four new compatible origins of replication intended to be part of plasmids for co-expression in *E. coli* as well as new expression systems, either constitutive (e.g. the *P_EM7_* promoter) or inducible by arabinose. Also, the new list contains a construct with the gadget *hok/sok* (see above). These genes encode a postsegregational killing device through which translation of a toxic gene (*hok*) is prevented by the *sok* RNA in such a way that cells that lose the plasmid are actively killed by the system (9).

### Honorary SEVA vectors

Another novelty in the list of available plasmids through the SEVA-DB is the incorporation of a separate catalog of constructs under the denomination/tab of SEVA-SIB collection. This is a series of plasmids contributed by the extended community of SEVA users in which most features of the standard are kept but some others miss one or more of the compositional rules and cannot thus be formalized as components of the official SEVA list. Many of them, however, are endowed with very useful cargoes that could certainly be of interest for the extended SynBio community. In particular, users can find in the SEVA-SIB collection the most relevant constructs of the so-called GeneGuard system for making plasmid vectors and host strains completely dependent on each other and thus increase the biosafety of the resulting recombinant materials (10). Another set of constructs of the SEVA-SIB list includes the Tn*5*-based mini-transposon-delivery vectors named pBAMD (11). These were designed both for insertion mutagenesis and for stable delivery of gene(s) of interest into the chromosome of diverse Gram-negative bacteria. The cargo site of the corresponding mini-transposons is identical to the default multi-cloning sequence of the canonical SEVA cargo (1), what eases the eventual transfer of constructs assembled in SEVA vectors to the genome of a target strain of choice. The pBAMD plasmids can be considered a beta-version of what will develop as a separate standard for transposon vectors (see below). Contributors to SEVA-SIB kindly offer their constructs for sharing through the SEVA database and plasmid repository. The various inputs of third parties to the SEVA-DB either in terms of materials or as bioinformatic resources are listed and kindly acknowledged in the tab *The SEVA Commonwealth*.

### Access to SEVA constructs

The SEVA-DB adheres to an Open Access practice that is the trademark of much of the Synthetic Biology community (12). This means that constructs of the SEVA-DB are freely distributed to academic researchers and other non-profit laboratories without requesting a material transfer agreement (MTA). In practical terms, the Laboratory that maintains the SEVA-DB is prepared to kindly ship at no cost for the solicitor up to three vectors in an ordinary small package through regular mail with the strains prepared as stabs in agar tubes. Requests for faster delivery or involving more than three items are passed to a handling agent, who may charge a managing fee for strain preparation and shipping. Broken-down requests to avoid this circumstance will be discarded. The method and the terms for adding new constructs to the SEVA collection are explained in detail in the earlier publication (1) and summarized in the DB webpage. Contributed SEVA vectors are in all cases verified by the curators of the collection and assigned a code that follows the rules above, or otherwise placed in the SEVA-SIB list. Users are kindly requested not to give (let alone publish) separate SEVA codes to constructs that are not explicitly officialized by the SEVA-DB managers. In case that some of the vectors develop into materials of commercial interest, users must check the intellectual property status of the corresponding biological parts, as the SEVA platform disclaims any carrier liability.

## ONGOING DEVELOPMENTS

### New functional segments and vector types

A substantial growth of the SEVA-DB and its matching vector collection is expected to occur in the near future. Work in progress includes the setup of a branch of SEVA plasmids for Gram-positive bacteria, a most desirable development in view of the comparatively low number of genetic tools available for microorganisms of this type other than *Bacillus subtillis.* It is noteworthy that SEVA plasmids with an RSF1010 origin of replication (e.g. pSEVA651) have been recently reported to function well in Mycobacteria (13) an offshoot of the Gram-positive family. But whether the same may work generally in further microorganisms of the kind remains uncertain. Other ongoing efforts are currently directed to elaborate a well-stuffed list of standardized heterologous expression systems that respond to diverse chemical inducers, metabolizable by the host or not. The favorite source of such systems is the wealth of regulatory nodes that control expression of pathways for biodegradation of recalcitrant and xenobiotic compounds of environmental bacteria (14). These are typically composed of four elements: a (i) relatively low-activity promoter that transcribes (ii) the gene of a regulatory protein that responds to a (iii) given inducer and which, once activated, triggers transcription initiation of a (iv) cognate promoter. A number of such standardized expression cargoes involving many of such regulators, e.g. AlkS (activated by *n-*octane), NahR (by salicylate), CprK (*o-*chlorophenolacetic acid) and ChnR (cyclohexanone) are well under way and will become soon a valuable addition to the vector panoply. A separate expansion of the repository deals with standards for either directed or random insertion of DNA of interest in the chromosome of target carriers. The literature contains many genetic tools to this end based on a variety of transposons, specifically Tn*5* (15), Tn*7* (16), Tn*10* (15) and the mariner transposon (17). Alas, as was the case with plasmid vectors, these tools are also afflicted by a chaotic lack of formatting that limit their application and the exchangeability of their parts. The above-mentioned pBAMD series of Tn*5*-based transposon vectors constitutes a good step toward subjecting this type of genetic tools to a serious standardization effort, but much still remains to be done.

### Describing the SEVA collection with synthetic biology open language (SBOL)

**Figure 3.**
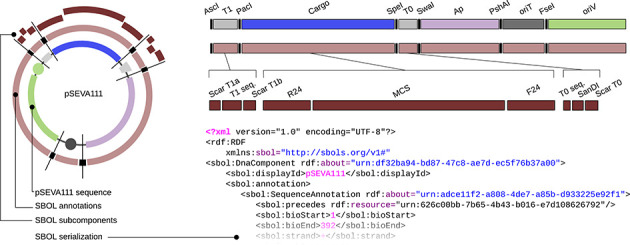
Details of the translation of pSEVA111 into SBOL format. The image to the left represents the breakdown of pSEVA111 (inner circle) in different sections until every single base in the sequence is assigned a role. This process is done hierarchically from up (components) to bottom (subcomponents). We first separate the sequence into ‘SBOL annotations’ (enzyme targets in black and functional modules in light brown) and second divide them into subunits when possible (dark brown). Names of all parts are shown at the right side of the figure. Terminator ‘T1’ is considered the whole sequence that goes from *Asc*I to *Pac*I and it is composed of three subcomponents: scars ‘ScarT1a’ and ‘ScarT1b’ (bases that do not correspond to the terminator itself but are there due to assembly imperfections), and ‘T1seq.’ which is the T1 sequence (in SBOL the labels ‘T1’ and ‘T1seq.’ refer to well differentiated segments and cannot be named with the same tag). In the same way, the ‘Cargo’ section is divided into: primer sites ‘R24’ and ‘F24’ and the multiple cloning site ‘MCS’. Terminator T0 is formed by the original sequence ‘T0seq.’, a target for ‘SanDI’ and another scar, ‘ScarTO’. The SBOL description is finally serialized as an RDF/xml file (see Supplementary File S1), a sample of which is pasted at the bottom of the figure.

**Figure 4.**
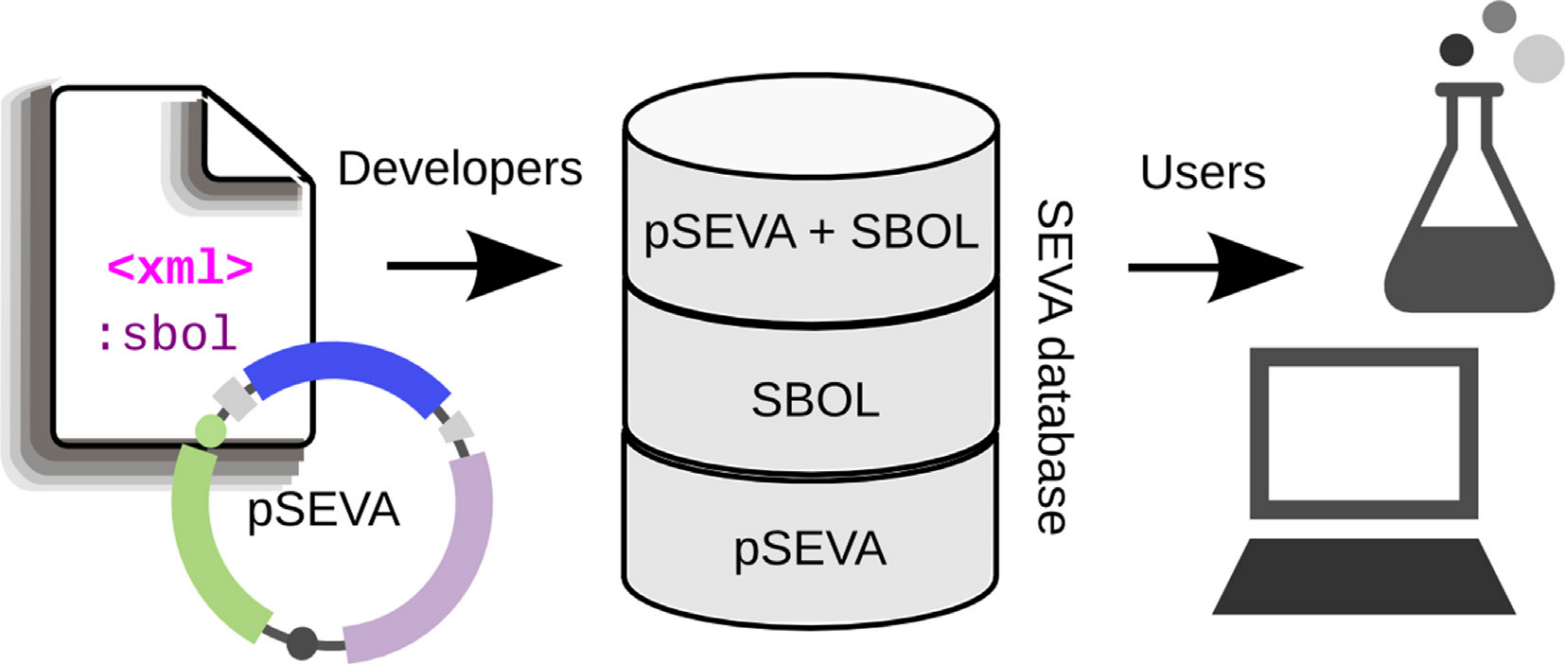
The SEVA database with the SBOL extension. The goal of the sketched flowchart is the attachment of an SBOL file to each SEVA vector in the collection and the use of that information for managing a queryable database. Developers and administrators will contribute by inputting and storing data using three levels: the repository of SEVA plasmids, the repository of their corresponding SBOL descriptions and a composite list of both items. End users will be able to retrieve that information to use it in the wet laboratory as well as in a previous *in silico* design stage.

The effort to standardize the physical and functional composition of the genetic tools that shape the SEVA collection would be in vain if we could not make the system compatible with former, current and future genetic engineering platforms. To this end, we pursue the representation of every significant functionality embedded in each of the vectors of the collection with the formalisms of the Synthetic Biology Open Language (SBOL, http://www.sbolstandard.org/). This is an open-source standard for *in silico* representation of genetic designs that, inter alia, allows exchanging designs, sending and receiving genetic circuits to and from biofabrication centers, facilitate their storage in repositories and embed genetic designs in publications (6–8). One example of SBOL description of plasmids of the SEVA collection is that of pSEVA111 using the cognate data format. The result is an XML file (Supplementary File S1) containing the annotation of the whole plasmid sequence where the functional segments are distinguishable, identifiable and accessible (Figure 3). A potential user could, for example, open the file using SBOL Designer (http://clarkparsia.github.io/sbol/), a software tool for creating and visualizing designs, and simply replace the origin of replication (R6K in pSEVA111) with the pBBR1 sequence in order to build pSEVA131 following the same SBOL standard. The provided demo file (pSEVA111.xml) has been validated using the Java library libSBOLj (https://github.com/SynBioDex/libSBOLj) and can be imported by software tools as a correct SBOL construction (Supplementary Figure S1). Ongoing efforts are committed to develop a query-able database of SBOL files (Figure 4), one for each SEVA vector plus isolated parts as antibiotic resistances or origins of replication. When designing a genetic circuit with a software tool, the user is able to import the carrier plasmid and embed the new design as the cargo segment. Therefore, the final sequence of the vector would be unequivocally annotated following standard descriptions (SBOL) and structures (SEVA) and ready to be shared or built. This would enable, for example, the synthesis of the newly defined cargo and the selection of a SEVA vector from the collection to integrate them both. As the SEVA vectors are widely used, the experimental information regarding their behavior under specific conditions is, at many cases, available and known. Taking advantage of that, we aim at enriching the SBOL files with important data about the ‘context’ of a vector so that the same user could choose between selecting either one plasmid or another depending on, for example, its copy number. This ‘context’ feature, which will complete the description of the system, is not included for now in the current SBOL, but the Developers Group are expected to release in the near future an updated version that fully supports extension (6).

## CONCLUSION

One key step toward transforming recombinant DNA technology into an authentic engineering discipline is the adoption of shared standards for easing biological design (2,18). Although the challenge, even the viability of the endeavor is plagued with difficulties (5,19) one facile starting point is the development of shared formats, designations and compositional rules for the genetic tools that are at the basis of a plethora of fundamental and biotechnological projects. The SEVA platform is a simple, robust and useful instrument to that end which has reportedly helped an active community of users in bringing Synthetic Biology to a suite of applications both in model bacteria (e.g. *E. coli*) and in industrially important cell factories (*Pseudomonas putida*; 20). While the ultimate destiny of genetic engineering is complete synthesis of any DNA sequence of interest (for which assembly vectors may not be necessary any longer), molecular tools for analysis and deployment of traits and genes of interest will still be required for a considerable period of time, in particular for addressing basic biological questions in non-model bacteria and for combining engineered properties with pre-existing biological qualities. As evidenced by the experience of the past two years, this is the area of action where the effort to maintain and further expand the SEVA platform deploys its full value. In the meantime, we will pursue the convergence of our platform with other vector standardization initiatives (12,21).

## SUPPLEMENTARY DATA

Supplementary Data are available at NAR Online.
